# The Standard of Preliminary Education

**Published:** 1911-06-17

**Authors:** 


The Hospital

A Journal of

The Medical Sciences and Hospital Administration.

New Series. No. 229, Vol IX [No. 1301, Vol. L.] SATURDAY, JUNE 17 1911

### The Standard of Preliminary Education.

A considerable ferment seems to have been created as the ultimate result of a report by the Director-General of the Naval Medical Service, acted upon by the General Medical Council. The Director-General, no doubt on mature reflection and with ample reason, felt constrained to bring officially to notice the fact that quite a number of candidates for admission to the Service of which he is the head display illiteracy of a kind which stamps their general education as imperfect. This complaint is, it is regrettable to admit, not a new one; it was made in 1890 and 1894 by the War Office, and in 1897, 1898, and 1899 by the Medical Boards of the Army, Navy, and Indian Medical Services. As a result of some of these complaints steps were taken by the General Medical Council to exact a higher standard of preliminary education, and certain examinations which seemed to have been conducted too laxly were struck off the list of those recognised for the purposes of registration as a student. The time has arrived, as the General Medical Council very properly recognises, when a further process of levelling up has become necessary; or, failing that, a weeding out of those examinations whose standard can be reached by men subsequently found unfit to receive commissions in His Majesty's Service afloat. As a first step towards this end the Executive Committee of the Council requested the Director-General to furnish a list of the examining bodies (in general education) responsible for the admission of the candidates stated to be deficient. The list supplied in answer to this request shows that out of fifteen candidates thus reported on in the last three batches of those seeking admission to the Naval Medical Service, four were sponsored by the University of Dublin and four more by the Royal University of Ireland. The College of Preceptors and the Educational Institute of Scotland accounted for two more each, and the remaining three had passed various (English) qualifying examinations. The report of the Executive Committee states that the majority of cases thus reported to the Council probably occur among those who have been prepared by cramming for an examination for which they are not qualified by real training. The system of examining and marking which permits candidates wanting in the essentials to pass with any degree of frequency is undoubtedly defective, and its employment by any responsible body is a breach of the entrusted duty.

The General Medical Council in November 1910 communicated to the authorities of several of the accepted preliminary examinations an intimation that their certificates might no longer be accepted for registration of medical study. It is difficult to see what other course the Council could adopt, since after all the proof of the pudding is in the eating. The various Boards replied for the most part in highly indignant vein; indeed, some of the letters were so tart that the Executive Committee in its present report feel obliged to point out that the action taken was in no way directed against the bodies conducting the examinations in question, but was concerned simply with the examinations characterised by the expert educational advisers of the Council as distinctly of the junior type. Most of the bodies mentioned do in fact conduct other and more suitable examinations recognised by the Council, which is clearly performing a plain duty in supervising the standard of preliminary education demanded of those whom it registers. If these examining corporations resent criticism, refuse to supply information, display hostility towards investigation, and decline to set their educational houses in order, they really cannot complain if, having been proved to pass candidates so illiterate as to be unable to spell, they are struck off the General Medical Council's list. It is apparent that some of them have done all, or nearly all, of these things; and in the interests of the profession at large we cannot do otherwise than express approbation of the General Medical Council's action.

The Medical Council may have been making itself unpopular with some of the more backward examining corporations, but it has done its duty; and we hope the hubbub created by its action will not distract attention from that fact and from the necessity for its constant and further vigilance in respect of these preliminary examinations in general education.

